# *Bartonella* transmission and gut microbiome dynamics in *Ceratophyllus sciurorum* fleas and their edible dormouse hosts (*Glis glis*)

**DOI:** 10.1186/s13071-026-07494-y

**Published:** 2026-06-11

**Authors:** Běla Klimešová, Karolina Volfová, Iva Hammerbauerová, Kristýna Dulavová, Petr Pajer, Kateřina Bundilová, David Modrý, Peter Adamík, Jan Votýpka

**Affiliations:** 1 Department of Parasitology, Faculty of Science, Charles University, Prague, Czechia; 2 Military Medical Agency, Military Health Institute, Prague, Czechia; 3 Department of Zoology, Faculty of Science, Palacký University, Olomouc, Czechia; 4 Institute of Parasitology, Biology Centre, Czech Academy of Sciences, České Budějovice, Czechia; 5 Department of Veterinary Sciences, Faculty of Agrobiology, Food and Natural Resources, Czech University of Life Sciences, Prague, Czechia; 6 Department of Botany and Zoology, Faculty of Science, Masaryk University, 625 00 Brno, Czech Republic

**Keywords:** *Bartonella*, Edible dormouse (*Glis glis*), Squirrel flea (*Ceratophyllus sciurorum*), Flea-borne transmission, Zoonosis, Microbiome, Transstadial perpetuation

## Abstract

**Background:**

The genus *Bartonella* comprises facultative intraerythrocytic bacteria capable of causing long-lasting bacteremia in their natural hosts, with zoonotic potential across multiple species. Rodents serve as important reservoirs for a broad diversity of *Bartonella* spp., with blood-feeding arthropods, particularly fleas, mediating transmission. Despite their frequent association with humans, the role of edible dormice (*Glis glis*) and their fleas (*Ceratophyllus sciurorum*) in *Bartonella* ecology remains poorly understood.

**Methods:**

We combined long-term ecological and epidemiological data with gut and body microbiome analyses of *C. sciurorum* to investigate the prevalence, diversity, host specificity, and transmission of *Bartonella* across flea life stages. The study was conducted over 6 years in a natural dormouse population. *Bartonella* detection and characterization were performed using multilocus PCR targeting *gltA*, *rpoB*, *ftsZ*, and ITS loci, bacterial cultivation on selective media, and long-read nanopore sequencing. Flea gut microbiomes were assessed in pooled and individual samples to determine the impact of *Bartonella* infection on microbial community structure. Transmission across flea life stages was evaluated by analyzing larvae, newly emerged adult fleas, and adults directly collected from dormice.

**Results:**

We observed a consistently high prevalence of *Bartonella* in both dormice and their fleas. Four species were identified: *B. gliris* and *B. grahamii* subsp. *shimonis* dominated, each represented by multiple genotypes, whereas *B. washoensis* and *B. bilalgolemii* were each detected in a single flea. Moreover, mixed infections of *B. gliris* and *B. grahamii* subsp. *shimonis* were frequent in both dormice and their fleas. Detection of *Bartonella* DNA in flea larvae and newly emerged adults indicates possible transstadial perpetuation. Flea gut microbiomes were highly variable but consistently dominated by *Bartonella* in infected fleas. In contrast, uninfected fleas exhibited more diverse communities, often enriched with *Staphylococcus* and other environmental or host-associated taxa. We, therefore, suggest that flea populations can maintain *Bartonella* over extended periods, even in the absence of continuous contact with vertebrate hosts.

**Conclusions:**

The dormouse–flea*–Bartonella* system represents a valuable natural model for studying flea-borne zoonoses. The high prevalence and persistent bacteremia in dormice of different ages indicate their role as suitable reservoirs for at least two *Bartonella* species. Flea populations are capable of sustaining *Bartonella* over long periods, and detections in immature life stages suggest continuity of infection across flea generations. Therefore, *C. sciurorum* appears to play an important role in maintaining *B. grahamii* subsp. *shimonis* and *B. gliris* in nature. Infection is consistently associated with pronounced restructuring of the flea gut microbiome, highlighting the ecological and microbial dimensions of vector-borne pathogen transmission. These findings underscore the importance of considering vector-associated microbial communities when evaluating disease dynamics and zoonotic risk in natural host–vector systems.

**Graphical Abstract:**

**Figure:**
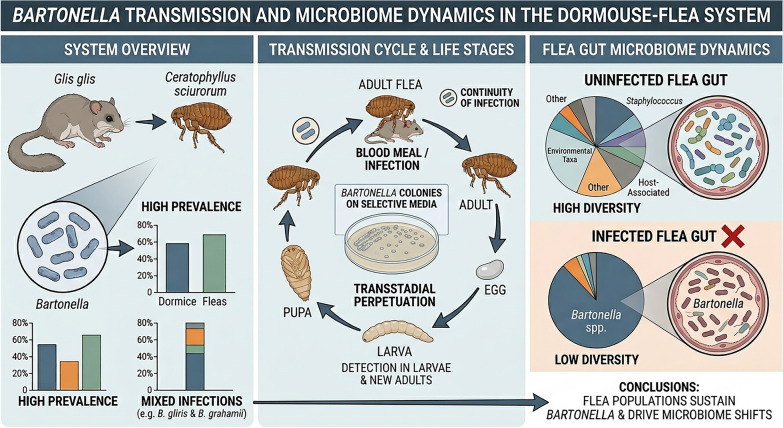

**Supplementary Information:**

The online version contains supplementary material available at 10.1186/s13071-026-07494-y.

## Background

*Bartonella* spp. are facultative intracellular bacteria that primarily reside within erythrocytes, causing long-lasting bacteremia with cyclic relapses, facilitated by reinfections of erythrocytes from endothelial cells [1]. In humans, bartonellosis can induce a broad spectrum of symptoms ranging from mild to life-threatening [2]. In contrast, infections in natural mammalian hosts are typically asymptomatic [3]. Over the last decades, several dozen new *Bartonella* species and subspecies have been described. The recognized host range, including rodents, ungulates, carnivores, humans, and even sea turtles, along with their global distribution, suggests that the number of described species will continue to grow [4–6]. Among mammalian hosts, rodents harbor the highest diversity of currently recognized *Bartonella* species, with prevalence typically ranging from 30–90% worldwide [7–11]. However, in Central Europe, most published studies have focused on the genera *Apodemus*, *Microtus, Myodes* (= *Clethrionomys*), and *Sciurus* (e.g., [12–17]), whereas data on other rodent taxa remain limited.

The edible dormouse (*Glis glis*), a nocturnal rodent of the Gliridae family, inhabits mainly deciduous and mixed forests in Europe [18, 19]. Dormouse populations depend strongly on the availability of seeds, particularly oak and beech, which constitute their main food source [20]. These rodents are obligatory hibernators, active only 3–5 months per year [21], and their long-term hibernation contributes to an unusually high longevity, up to 9 years [22, 23]. Reproduction is limited, with most females breeding only once or twice in their lifetime, typically in mast seeding years when resources are abundant [24, 25].

The potential of dormice to transmit pathogens to domestic animals and humans remains poorly understood [26]. Historically used as a food source and still hunted in some regions [18, 27], dormice frequently enter human dwellings and inhabit roofs or areas near rural houses and cottages, seeking shelter and food [28–30]. Due to their high levels of infestation by fleas and ticks, edible dormice are considered potentially important reservoirs of zoonotic pathogens transmitted by these vectors [26, 31], including *Borrelia burgdorferi* s.l. [32].

Blood-feeding arthropods harbor complex microbial communities that can profoundly influence their biology, vector competence, and interactions with pathogens transmitted by them [33, 34]. Evidence from mosquitoes, ticks, and fleas indicates that the gut microbiome may modulate pathogen acquisition, persistence, and transmission through competitive, synergistic, or immune-mediated mechanisms [35–37]. Despite this, the microbiome of flea vectors transmitting *Bartonella* spp. remains poorly characterized, with conflicting reports about the influence of *Bartonella* [35, 38]. Understanding the baseline composition and variability of flea microbiomes in both adults and larvae is, therefore, essential for modeling *Bartonella* transmission dynamics [11, 39, 40].

The squirrel flea *Ceratophyllus sciurorum* (Siphonaptera: Ceratophyllidae) is a common ectoparasite of arboreal rodents in Europe, particularly dormice and squirrels [41], and is frequently infected with *Bartonella*, making it an ideal model for studying interactions between flea-associated microbiota and *Bartonella* infections. To date, no detailed microbiome analysis of *C. sciurorum* has been published.

Despite the recognized importance of fleas as (at least suspected) vectors of *Bartonella* spp., the ecological mechanisms governing *Bartonella* persistence, diversity, and transmission in natural rodent–flea systems remain insufficiently understood. In particular, long-term data integrating host population ecology, vector infection dynamics, and pathogen diversity are scarce, limiting our ability to assess the stability and epidemiological relevance of flea-borne *Bartonella* cycles under natural conditions.

The primary aim of this study was, therefore, to characterize the long-term ecological and epidemiological dynamics of *Bartonella* spp. in a natural host–vector system composed of the edible dormouse (*Glis glis*) and its dominant flea ectoparasite, *Ceratophyllus sciurorum*. Using a 6-year dataset from a well-monitored dormouse population, we focused on *Bartonella* prevalence, species and genotype diversity, host specificity, temporal stability, and possible transmission across flea life stages, including infection persistence.

As a secondary and complementary objective, we explored the microbiome of *C. sciurorum* to provide an ecological context for *Bartonella* transmission within the flea vector. Rather than testing causal effects of the microbiome on pathogen acquisition or transmission, this component aimed to describe baseline microbiome composition, inter-individual variability, and broad associations between *Bartonella* infection status and microbial community structure. By integrating microbiome data with epidemiological observations, we aimed to determine whether consistent microbial patterns are associated with *Bartonella* infection in fleas under natural conditions.

Together, this integrative approach allows us to place *Bartonella* occurrence within a broader ecological framework, highlighting how long-term host–vector interactions and vector-associated microbial communities jointly shape flea-borne bartonellosis in nature.

## Methods

### Collection of samples: dormice and fleas

Edible dormice (*Glis glis*) and their fleas were regularly sampled several times per season over a 6-year period (2016–2021; Table 1) at an established nest box population [42], with some fleas collected for microbiome studies even after this period. The study was conducted in a mixed woodland near the municipality of Dlouhá Loučka, Olomouc district, Czech Republic (Fig. S1).

**Table 1:** Sampling overview of dormouse blood, fleas from dormice, and fleas from nests collected over a 6-year period (2016–2021)

Years	Dormice	Fleas from dormice	Fleas from nests
2016	16	N/A	N/A
2017	61	102	N/A
2018	39	102	N/A
2019	63	222	10
2020	19	35	65
2021	3	49	112
Overall	201	510	187

The dormice were removed from the nest boxes, which they used as resting sites, and anesthetized via intramuscular injection of ketamine (20 mg/kg)/medetomidine (0.2 mg/kg). Blood was sampled from the cranial vena cava using a sterile hypodermic needle and collected into microtubes containing either EDTA (for cultivation) or ethanol/SDS + EDTA (for DNA extraction). After sampling, the dormice were revived from anesthesia using intramuscular injection of atipamezole (Revertor, 0.2 mg/kg) and returned to their original nest boxes.

Each dormouse was sexed, aged, weighed, and identified with a PIT tag using a standardized handling protocol [43, 44]. A total of 202 blood samples from 201 dormice were obtained, since only one dormouse was sampled twice. In addition, the animals were brushed to collect ectoparasites (*n* = 510). In a subset of dormouse nests, nesting material was also sampled. Nest material was placed in plastic bags, stored at 5 °C, and live fleas were later collected by handpicking (*n* = 187). Fleas sampled directly from dormice were stored either in 1.5-mL plastic tubes filled with 70% ethanol (for PCR analysis only; stored at − 20 °C) or in empty tubes (for dissection, cultivation, and PCR; stored at 5 °C for up to 1 week).

Although dormouse blood was sampled during six summer seasons, fleas from dormice were not collected until 2017, and nest material for flea inspection was first collected in 2019 (see Table 1 for a sample overview). Nest material collected in 2021 was used to investigate the potential for vertical transmission of *Bartonella* in fleas.

### DNA extraction, PCR amplification, sequencing, and bioinformatic processing

Live fleas were killed by freezing, then immersed in 70% ethanol for surface decontamination and briefly washed three times in sterile phosphate-buffered saline (PBS) solution, following the guideline from Gutiérrez et al. [45]. All fleas were morphologically identified under a stereomicroscope using the identification key of Rosický [41]. The vast majority (> 95%) were confirmed as *Ceratophyllus* (*Monopsyllus*) *sciurorum*, and all subsequent analyses were conducted exclusively with this species. Sex was determined for 410 fleas, with females making up 56% of all fleas examined.

For the study of *Bartonella* prevalence, fleas were individually homogenized using sterile pestles in 100 μL of SIMB medium (Schneider’s Insect Medium, Sigma-Aldrich, supplemented with 10% fetal calf serum, 5% sucrose, and 0.1% amphotericin B), following the protocol recommended by Gutiérrez et al. [45] for *Bartonella* cultivation. The medium containing dissolved sucrose was filtered through a 0.22-µm Millex filter into a fresh tube before use. For samples processed for DNA extraction only, fleas were homogenized in sterile PBS (Fig. S2).

Dormouse blood (in ethanol or SDS + EDTA) and homogenized whole-flea samples were used for DNA extraction with the DNeasy Blood and Tissue Kit (QIAGEN) according to the manufacturer’s instructions. The final elution step was modified by replacing the elution buffer (EB) with a 1:1 mixture of EB and PCR-grade water, and the total elution volume was 100 µL.

Subsequently, (nested) PCR assays were performed targeting four *Bartonella* loci: citrate synthase (*gltA*, 350 bp), the β-subunit of RNA polymerase (*rpoB*, 400 bp), a cell division protein (*ftsZ*, 515 bp), and the 16S–23S rRNA internal transcribed spacer (ITS, 200–350 bp). PCR conditions followed Majerová et al. [16], (Table. S1–2). Given the high sensitivity of nested PCR to cross-contamination, strict precautions were implemented: individual steps of the workflow (DNA extraction, PCR setup, and post-PCR processing) were conducted in physically separate rooms, PCR workstations (PCR boxes) were used, and aerosol-resistant filter tips were employed throughout. ITS PCR products were generally not further processed, as electrophoresis allowed discrimination of the two most common *Bartonella* species based on product size (~ 220 bp and ~ 320 bp). Positive *gltA*, *rpoB*, and *ftsZ* PCR products were purified using ExoSAP-IT^™^ (Thermo Scientific), sequenced, and checked in Geneious Prime, where further processing, including sequence trimming and genotype detection, was performed. Sequences were compared against the NCBI GenBank database for species-level identification. As all detected genotypes belonged to well-characterized *Bartonella* species and no novel species were identified, phylogenetic analyses were considered unnecessary.

For the flea microbiome study, two independent experiments were conducted, namely, those using pooled samples and individual fleas. All fleas in both experiments were washed once in ethanol and twice in PBS (phosphate-buffered saline) before processing, and dissection was carried out on sterile microscopy slides with tools sterilized by ethanol and fire. In the first experiment, 60 adult fleas (30 males and 30 females) were dissected to separate the midgut and hindgut. The respective gut tissues were then pooled in groups of five individuals. In addition, one pool of five surface-washed flea larvae was prepared.

In the second experiment, 20 individual adults were analyzed separately to capture inter-individual variability. For this experiment, the fleas were dissected into two parts, namely, the whole gastrointestinal tract (GIT) and the rest of the body. Total DNA was extracted from both pooled and individual samples. A drop of PBS buffer handled with dissection tools was used as a negative control to rule out contamination during processing for each batch of samples (6 in total). For the second experiment, an additional control sample consisting of nest soil was included. Long-read sequencing libraries were prepared and sequenced using the 16S Barcoding Kit SQK-16S024 (Oxford Nanopore Technologies) utilizing the 27F (5′-AGAGTTTGATCCTGGCTCAG-3′) and 1492R (5′-TACCTTGTTACGACTT-3′) universal 16S rRNA primers. Some samples yielded low read numbers and were excluded from downstream analyses. Raw reads were filtered and processed using the PRONAME pipeline [46] to remove low-quality reads (*Q* score < 15), trim primers, remove chimeras, and generate operational taxonomic units (OTUs) with vsearch (clustering threshold of 0.95) [47]. Contaminant OTUs were removed using the R package decontam with the frequency and prevalence methods [48]. Taxonomic assignment to the genus level was performed using the SILVA database [49]. Unassigned OTUs, as well as OTUs assigned as chloroplasts or mitochondria were excluded from downstream analyses.

### Culture-based analyses

Cultivation of *Bartonella* spp. was performed as described previously [50]. Briefly, the cultivation medium SIMB was added to blood (*n* = 81) or flea homogenate samples (*n* = 122; 79 from dormice and 43 from nests), which were subsequently inoculated onto chocolate agar plates. Plates were sealed with Parafilm to prevent desiccation and incubated at 37 °C in an atmosphere enriched with 5% CO_2_ for up to 6 weeks. For *Bartonella* species determination, up to 32 colonies per original sample were analyzed using single-step direct PCR targeting either the ITS region or a partial fragment of the *gltA* gene (corresponding to the first step of the nested PCR protocols).

For the microbiome study, 24 pooled gut homogenates (midguts and hindguts separately) from the first experiment were inoculated onto five types of selective and non-selective culture media: blood agar, chocolate agar, MacConkey agar, mEnterococcus agar, and tryptic soy agar (TSA) and incubated at 25 °C for up to 2 weeks. Bacterial colonies were selected based on morphological characteristics, subcultured, and identified using PCR targeting the 16S rRNA locus using universal primers Bakt_341F (5′-CCTACGGGNGGCWGCAG-3′) and Bakt_805R (5′-GACTACHVGGGTATCTAATCC-3′) according to Herlemann et al. [51]. In parallel, samples were screened for *Bartonella* DNA using nested PCR targeting the *gltA* gene.

### Transmission of *Bartonella* between different flea life stages

Experiments on the transmission of bartonellae within fleas were conducted using dormouse nests collected either during the active season of dormice (summer) or during winter, when dormice were overwintering outside the boxes. Upon transport to the laboratory, the nest material, consisting of leaves and debris, was thoroughly and repeatedly examined for adult fleas and larvae. Larvae were collected using soft entomological forceps. Only the last larval instar, which is easily distinguishable by eye, was collected; however, all specimens were additionally checked under a stereomicroscope to confirm identification. Nest material was screened approximately once per week, typically over a period of 1 month.

Up to 10 adult fleas and larvae per nest (except for one nest with 22 adult fleas, Table. S3) were used for DNA extraction followed by PCR analysis. After this procedure, the nests were stored in plastic bags at room temperature and checked weekly for newly emerged adult fleas over a 6-week period. The newly emerged fleas (which had no direct contact with potentially infected dormice) were used for PCR targeting ITS and, in some cases, also for cultivation. Up to 25 newly emerged adult fleas per nest were collected and processed (Table. S3).

### Statistical data analysis

*Bartonella* prevalence (binomial data) was analyzed using generalized linear mixed-effects models (GLMMs) applied separately to (i) individual dormice, (ii) fleas collected directly from dormice, and (iii) fleas collected from dormice nests.

For the dormice, their age (adult or young), body weight, sex, and sampling year were included as fixed effects. For the fleas collected directly from dormice, sampling year, and flea sex were modeled as fixed effects, with host individual included as a random effect; analyses were conducted both with and without flea sex due to missing data in some samples. For fleas collected from dormice nests, the sampling year was modeled as a fixed effect and nest identity as a random effect. Year was treated as a categorical factor in all models.

The presence of individual *Bartonella* species (*B. gliris* and *B. grahamii* subsp. *shimonis*) was analyzed using general linear mixed-effects models, testing reciprocal associations between the two species while accounting for additional predictors. Fixed effects included host sex, age, weight, and sampling year for dormice, and sampling year for fleas, with host identity (fleas collected from dormice) or nest identity (fleas collected from nests) included as random effects where appropriate.

Differences in *Bartonella* prevalence among three flea life stages (blood-feeding adult fleas, larvae, and newly emerged adult fleas with no contact with dormice) were assessed using a general linear mixed-effects model with nest identity as a random effect.

The effect of each predictor was evaluated using a Type II Wald *χ*^2^ test [52]. Statistical significance was determined at *P* < 0.05. All statistical analyses were performed using the statistical computing environment R version 4.3.3 [53]. The package lme4 [54] was used to test the models, and ggplot2 [55] was used for visualization.

Microbiome analyses were conducted in QIIME2 [56] and RStudio [57]. Alpha diversity metrics were calculated in QIIME2 after normalizing samples by rarefaction (threshold for pooled samples = 255; threshold for individual fleas = 715). Beta diversity was calculated using the weighted UniFrac metric [58], and principal coordinate analysis graphs were prepared with ggplot2 [55].

## Results

*Bartonella* infections were detected in blood samples from dormice and their fleas using a multi-method approach, combining PCR targeting four *Bartonella*-specific loci (*gltA*, *rpoB*, *ftsZ*, and ITS) with bacterial cultivation, and applied to a total of 204 samples (Table. 2). The overall *Bartonella* prevalence in dormice samples, as determined by all methods combined, was 88% (180/204). Among individual methods, the highest prevalence was detected by ITS PCR (76%; 155/204), while the lowest was by *ftsZ* PCR (44%; 90/204).

**Table 2:** *Bartonella* prevalence detected in sampled dormice (blood samples), fleas collected directly from dormice, and fleas collected from nests, tested for *Bartonella* presence using PCR (targeting four loci: *gltA, rpoB, ftsZ*, and ITS) and bacterial cultivation on agar plates (performed on a subset of samples)

Detection method	Detected prevalence (positive samples/total number of analyzed)		
	Dormice	Fleas from dormice	Fleas from nests
*gltA*	71% (145/204)	82% (418/510)	38% (59/155)
*rpoB*	65% (133/204)	76% (388/510)	39% (60/155)
*ftsZ*	44% (90/204)	65% (332/510)	28% (43/155)
ITS	76% (155/204)	83% (423/510)	29% (45/155)
Cultivation	60% (49/81)	22% (17/79)	7% (3/43)
Overall	88% (180/204)	90% (458/510)	40% (62/155)

In fleas sampled directly from dormice, the overall *Bartonella* prevalence was 90% (458/510), with ITS again detecting the highest prevalence (83%; 423/510), whereas cultivation yielded the lowest detection rate (22%; 17/79). In contrast, flea samples collected from the same nests exhibited a substantially lower overall *Bartonella* prevalence (40%; 62/155), with *rpoB* detecting the highest prevalence (39%; 60/155) and cultivation again showing the lowest detection rate (7%; 3/43). The substantially reduced detection success of cultivation in flea samples was attributable to a high contamination rate, as 38% of samples (22/81 blood and 55/122 flea samples) were overgrown by contamination, preventing accurate assessment of *Bartonella* presence.

### Prevalence of detected bartonellae

**Fig. 1:**
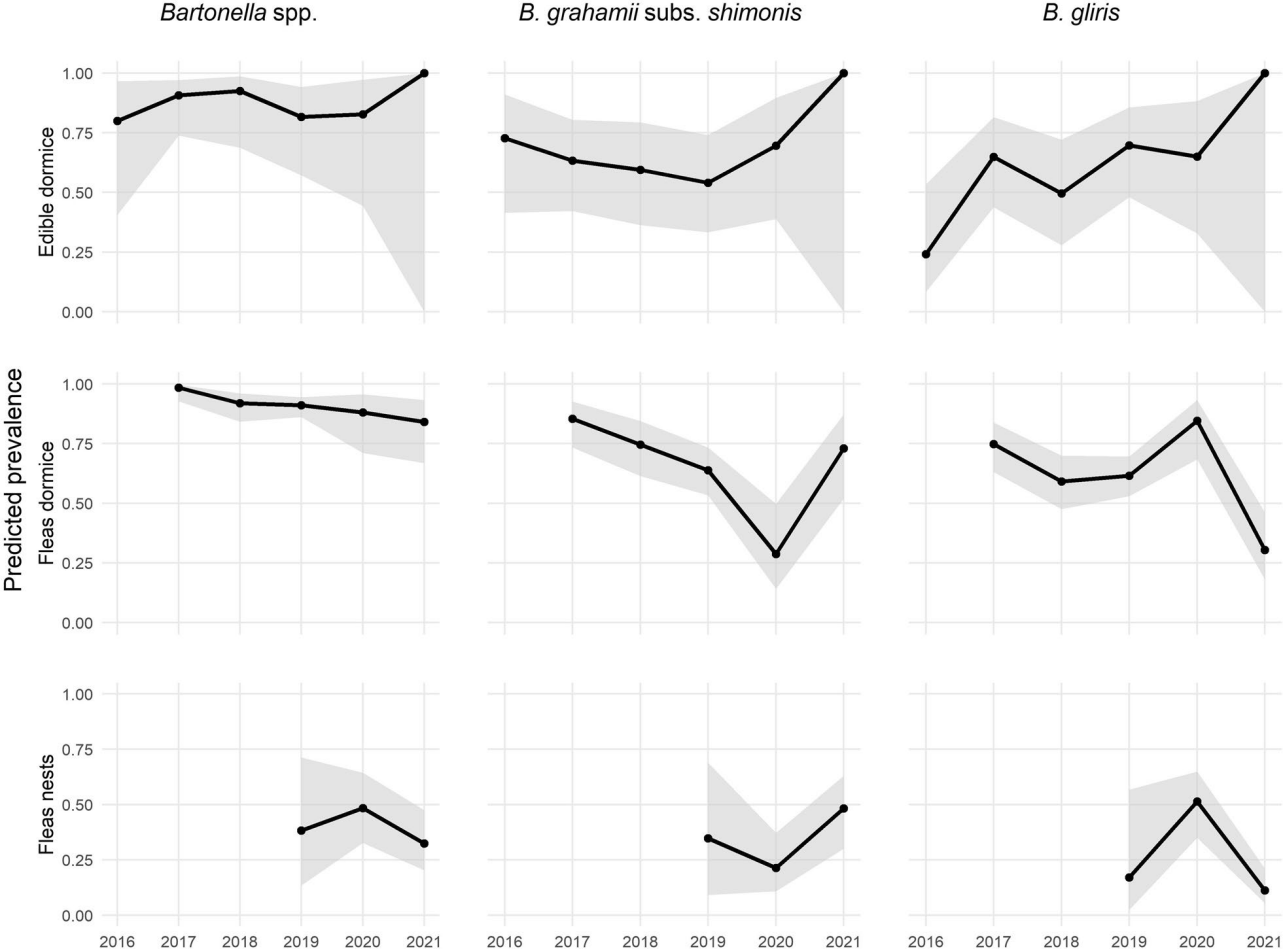
Overall detected prevalence and estimated occurrence of *B. gliris*, *B. grahamii* subs. *shimonis*, and their combined prevalence (reported as *Bartonella* spp.) throughout the years 2016–2021 in dormice blood samples, fleas collected directly from dormice, and fleas collected from dormice nests. The shaded area shows the 95% confidence intervals

The influence of multiple variables—host age, weight, sex, and year—on *Bartonella* spp. prevalence in dormice blood samples was examined, but none of these variables had a statistically significant effect (*P* > 0.05; Table. S4, Fig. 1). In flea samples collected directly from dormice, the effects of year and sex on *Bartonella* prevalence were analyzed, whereas in flea samples collected from dormice nests, only the effect of sampling year was tested. Although the year alone did not have a significant effect in either group (*P* > 0.05), a statistically significant effect of sampling year was detected in the model for fleas from dormice when sex was included as a predictor (Table. S4).

### Detected *Bartonella* species

In total, four distinct *Bartonella* species were detected. *Bartonella washoensis* was identified in a single flea sample by all four PCRs, with > 99% sequence similarity to previously published GenBank sequences across all targeted genes and ITS region. Another rare finding, detected in a single flea, was represented by a *gltA* sequence showing 100% identity to *Bartonella bilalgolemii*. No additional loci were successfully amplified for this sample, as all other PCR assays were negative.

In contrast, *Bartonella gliris* and *Bartonella grahamii* subsp. *shimonis* were frequently detected in both dormice and fleas, with multiple genotypes identified (Table. S11). Three predominant genotypes of *B. grahamii* subsp. *shimonis* (g1–3) and two genotypes of *B. gliris* (g1–2) were successfully cultivated and, after several passages [see 50 for details], their clones were fully characterized across all four PCR-targeted loci using long amplicons from the first step of nested PCR. Moreover, for two genotypes of each species (g1–2) originating from dormice, whole-genome sequencing was performed, resulting in the description of novel (sub) species [50]. Detection of individual genotypes in non-cultivated samples was subsequently assessed in some cases using short nested-PCR products, based on diagnostic nucleotide substitutions or deletions (in the ITS region) (Figs. S9 and 10).

An additional *gltA* genotype of *B. grahamii* subsp. *shimonis* (g4), differing by a single nucleotide substitution, was identified in one dormouse. Similarly, four additional *B. gliris* genotypes (g3–6), based on *gltA* and *rpoB* sequences with single nucleotide substitutions, were detected in one dormouse (g3) and in three fleas (g4–6). None of these rare variants was supported as a distinct genotype at other loci. Thus, dormice hosted four genotypes (g1–4) of *B. grahamii* subsp. *shimonis*, of which only g1–3 were detected in fleas. Conversely, for *B. gliris*, five genotypes (g1–2, 4–6) were detected in fleas, whereas only three (g1–3) were found in dormice. Genotypes g1–3 of *B. grahamii* subsp. *shimonis* and g1–2 of *B. gliris* were common and largely consistent across years and host groups (Fig. S3), whereas all remaining genotypes were rare and detected in single samples only.

In dormice, only the occurrence of *B. gliris* varied significantly among years (Fig. 1), whereas the prevalence of *B. grahamii* subsp. *shimonis* was negatively correlated with host body weight (Table. S5). In both fleas collected from dormice and those collected from nests, the occurrence of *B. gliris* and *B. grahamii* subsp. *shimonis* varied significantly among years (Fig. 1). In fleas from nests, the occurrence of each species was also associated with that of the other species (Table. S5).

Mixed infections with *B. gliris* and *B. grahamii* subsp. *shimonis* were detected in a high proportion of dormice (56%) and fleas collected from dormice (57%), and in a lower proportion of fleas from nests (27%). Mixed infections were identified based on (i) clear double peaks in sequence chromatograms at individual loci (*gltA*, *rpoB*, *ftsZ*), (ii) differences in amplicon size visualized by electrophoresis (ITS), and/or (iii) concordant or partially discordant results among PCR loci and between PCR and cultivation, indicating the presence of multiple *Bartonella* species within a single sample. Overall, multilocus PCR results and cultivation detected mixed infections in 53% of samples, compared with lower detection rates by individual methods: *gltA* (24%), *rpoB* (2%), *ftsZ* (32%), ITS (32%), and cultivation (26%) (Table. S6).

### Detection of *Bartonella* in different flea life stages

**Fig. 2:**
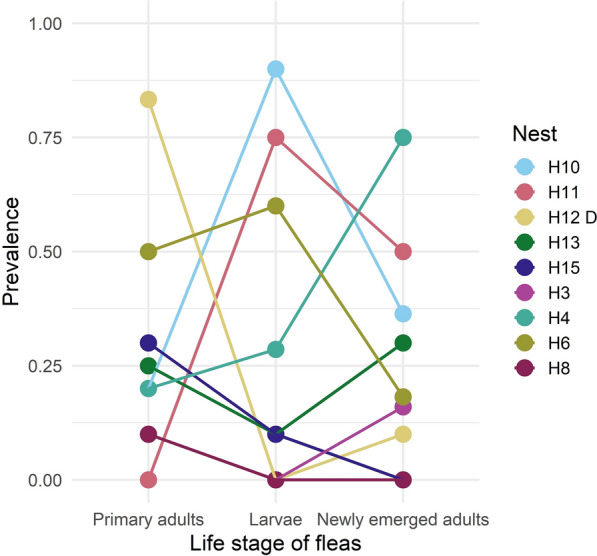
*Bartonella* prevalence in three life stages of fleas (primary adults, larvae, and newly emerged fleas). *Bartonella* prevalence in the eight nests, where all three flea life stages were present, with each nest represented by a separate color

Of the 17 nests processed, three were completely flea-free, and in two nests no newly emerged adult fleas and/or larvae were found; these five nests were, therefore, excluded from further analyses. *Bartonella* DNA was exclusively detected by ITS PCR, resulting in positivity rates of 31% (33/106) in primary adult fleas, 30% (22/73) in larvae, and 17% (22/126) in newly emerged adult fleas (Fig. 2). Differences in *Bartonella* prevalence among the three developmental stages were evaluated using a generalized linear mixed-effects model (GLMM), with nest ID included as a random effect and flea stage as a fixed effect. The effect of flea stage was not statistically significant (type II Wald *χ*^2^ = 2.69; *df* = 2; *P* = 0.26).

In total, homogenates of 31 newly emerged adult fleas, prepared as described above, were used for bacterial cultivation. Twenty-three samples were overgrown with contamination and could not be evaluated, and the remaining eight samples were *Bartonella*-negative.

### Flea gut microbiome composition and its association with *Bartonella* infection

A total of 464 colonies, selected based on colony morphology from cultured plates, were identified by 16S rRNA gene sequencing. The suitability of individual media for bacterial growth reflected the number of colonies recovered: the highest number was obtained from Tryptic Soy Agar (*n* = 154), followed by blood agar (*n* = 126) and chocolate agar (*n* = 115). In contrast, only 50 colonies were isolated from mEnterococcus agar, and the fewest from MacConkey agar (*n* = 19). Several operational taxonomic units (OTUs) grew on most of the media used; for example, three prevalent taxa—*Staphylococcus* OTU 1, *Mammaliicoccus* OTU 1, and *Brevibacterium* OTU 1—were detected across multiple media (Fig. S4).

In total, 77 OTUs were identified, including 45 recurrent and 32 unique OTUs. These OTUs were assigned to 35 bacterial genera, five of which were either unclassified or annotated as uncultured. *Staphylococcus* was the most abundant genus, accounting for over 30% of all sequences and comprising the highest number of OTUs (11). Other genera with relatively high OTU richness included *Arthrobacter* (7), *Brevibacterium* (5), and *Microbacterium* (5). Based on total sequence counts (i.e., number of colonies analyzed), the most abundant genera after *Staphylococcus* were *Brevibacterium*, *Mammaliicoccus*, and *Brachybacterium* (Table. S7).

Although cultivation on different media provided informative insights, nanopore sequencing revealed the full extent of bacterial diversity and, importantly, interactions with *Bartonella*. The gut microbiome of *Ceratophyllus sciurorum* showed high variability among samples. In the first experiment, 1429 OTUs were detected, and in the second, 1250. After decontamination (exclusion of 4 and 9 OTUs, respectively) and removal of unassigned OTUs or OTUs assigned as mitochondria or chloroplasts, 1419 OTUs (85 genera) from pooled samples and 1232 OTUs (57 genera) from individual fleas remained for downstream analyses. Read counts and numbers of taxa detected are included in Table S9.

**Fig. 3:**
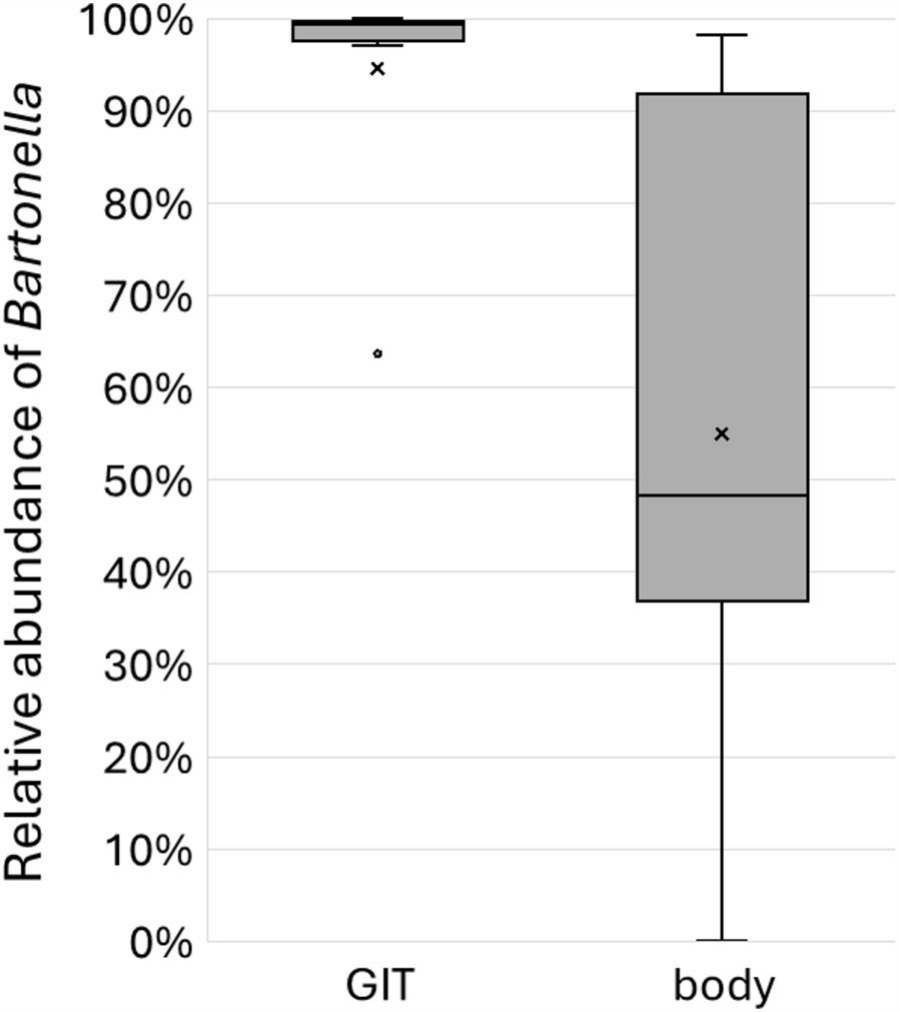
Relative abundance of *Bartonella* compared to other bacteria in gastrointestinal tract (GIT) and body samples of individual *Bartonella*-positive fleas. The mean value is marked with × , and one outlier sample with ●

Across both experiments (pooled gut samples and individual flea gut analyses), the bacterial communities were consistently dominated by Proteobacteria and Firmicutes. No *Rickettsia* or *Wolbachia* was detected. In *Bartonella*-negative fleas, the dominant taxon was *Staphylococcus*, consistent with cultivation results. Due to the design, *Bartonella* abundance in pooled samples was inconsistent; however, in PCR-positive samples, it was the dominant taxon (Fig. S5). No significant differences were observed between the hindgut and midgut composition. In pooled samples, only pools from female fleas collected in summer were strongly *Bartonella*-positive. These three samples, in which *Bartonella* was detected with PCR as well as metabarcoding showed a markedly different composition from *Bartonella*-negative or weakly positive pools, likely representing pools of several infected fleas (Fig. S6). Testing of individuals revealed *Bartonella* as the primary component in the digestive tract of infected fleas, accounting for the majority of reads and showing a clear similarity in the gut bacterial composition of infected fleas (Figs. S7–8). Alpha diversity metrics for each sample from both experiments are described in Table S10. Samples from flea larvae and guts of individual *Bartonella*-negative fleas contained too few read counts after quality filtering and decontamination and were not included in analyses. *Bartonella* DNA was also found in flea bodies, in smaller but still significant amounts (Fig. 3). In individual fleas, *Bartonella* was detected in both males and females, consistent with PCR results.

**Fig. 4:**
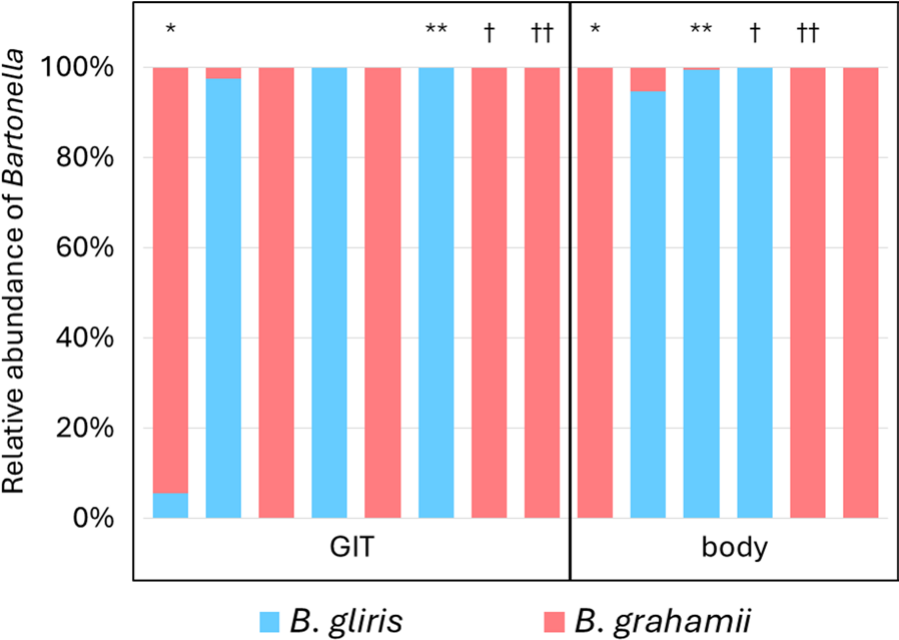
Relative abundance of the two *Bartonella* species in individual flea samples calculated from read counts. Samples that came from the same individual are marked by * or †

Two species of *Bartonella* were detected: *B. gliris* and *B. grahamii*, consistent with PCR detection. In most fleas, only one species was found, consistent with PCR results in nest fleas. If both *Bartonella* species were present, one was clearly dominant (Fig. 4).

## Discussion

### Diversity of detected bartonellae

In the present study, four *Bartonella* species were detected in dormice and their fleas. While *B. washoensis* and *B. bilalgolemii* were each detected in only a single flea specimen, *B. gliris* and *B. grahamii* subsp. *shimonis* were widely distributed across the analyzed samples and were represented by multiple genotypes. However, only two and three genotypes, respectively, were successfully cultivated and repeatedly detected across multiple samples. Notably, both *B. washoensis* and *B. grahamii* have been previously reported to have zoonotic potential [59, 60].

*Bartonella washoensis* is primarily associated with sciurid rodents (e.g., [61]), but it has also been reported in European hedgehogs (*Erinaceus europaeus*) in Czechia [16], dogs in the USA [62], and humans in both the USA and Germany (e.g., [14]). *Bartonella bilalgolemii*, identified based on a short *gltA* sequence (with other PCR assays and cultivation yielding negative results), represents another rodent-associated species, with members of the genus *Apodemus* as known hosts, namely, *A. uralensis* in Turkey (PP965719), *A. flavicollis* in Sweden (AY454539), and *Apodemus agrarius* in Croatia (KT452905) [63, 64]. Its detection in a dormouse flea suggests either flea exchange between syntopic *Apodemus* and *Glis* hosts or a possible spillover from *Apodemus*-associated hosts.

The *B. grahamii* species complex has been reported from a wide range of rodent hosts across Asia, Europe, and North America and is considered one of the most prevalent *Bartonella* taxa in rodents [9, 65]. While the recently described subspecies *B. grahamii shimonis* has been identified in multiple host genera (*Glis, Sciurus, Erinaceus*) in Czechia, *B. gliris* has so far been exclusively associated with the edible dormouse [16, 50].

A total of ten different genotypes were detected in fleas (three of *B. grahamii* subsp. *shimonis*, five of *B. gliris*, and one each of *B. washoensis* and *B. bilalgolemii*), whereas six genotypes were found in dormouse blood samples (four of *B. grahamii* subsp. *shimonis* and three of *B. gliris*). Notably, two genotypes detected in dormouse blood were novel and found exclusively in this sample type. The difference in detected genotype richness may be influenced by the substantially higher number of fleas analyzed (697) compared to dormice (201). As seven of the twelve genotypes, including both rare species, were each detected in only a single sample, the observed diversity difference may also partly reflect stochastic effects. Nevertheless, a higher *Bartonella* genotype diversity in fleas than in vertebrate hosts has been reported previously, for example, in *Gerbillus andersoni* in Israel, where six abundant genotypes were detected in fleas but only two in rodents [66]. These findings support the concept that mammals are exposed via fleas to a broad spectrum of *Bartonella* species/genotypes, which does not always result in detectable bacteremia [67].

The host specificity of blood-sucking vectors can influence *Bartonella* diversity in vertebrate hosts. Dormice may acquire fleas from different ecological contexts during foraging or nesting in sites previously occupied by birds or other rodents, and they are known to encounter diverse flea communities [41]. However, the dominant flea of edible dormice is the red squirrel flea, *Ceratophyllus* (*Monopsyllus*) *sciurorum* [26, 68, 69], which also parasitizes other small mammals, nesting birds, and occasionally humans [41, 61, 70]. Given the close ecological association between dormice and squirrels (e.g., *Sciurus vulgaris*), [61, 68], interspecific exchange of *Bartonella* spp. is plausible. While *B. grahamii* subsp. *shimonis* has been detected in both dormice and squirrels (provisionally designated as *Bartonella* sp. SCIER [16]), *B. gliris* has so far been reported exclusively from edible dormice, despite extensive screening of squirrels, including in Czechia (e.g., [14, 16, 61]).

Previous studies have reported high polymorphism in *B. grahamii*, with a pronounced geographical structure and greater diversity in Asia than in Europe [9, 71]. For instance, only four *B. grahamii* genotypes have been described across several rodent species, including *Apodemus agrarius* and *Myodes* (= *Clethrionomys*) *glareolus*, from multiple localities in Lithuania [72]. In contrast, this study detected four genotypes within a single locality and a single rodent species. This observation, however, may partly reflect the high-resolution approach used, combining cultivation and multilocus PCR across four genetic loci.

### Prevalence and dynamics of *Bartonella* infection

Whereas the overall prevalence of *Bartonella* spp. in dormice and fleas collected directly from dormice was nearly identical (88% and 90%, respectively), prevalence in fleas collected from dormice nests was substantially lower (40%). Because flea infection is considered lifelong [73, 74], these differences suggest that most nest-derived fleas had either not yet taken an infective blood meal or had not acquired infection via transstadial perpetuation .

Dormouse blood was sampled over 6 years, and no significant seasonal differences in *Bartonella* prevalence were observed. Similarly, no temporal changes in prevalence were detected in fleas sampled from dormice or nests, which were collected over 5 and 3 years, respectively. In contrast, studies on *Myodes glareolus, Apodemus flavicollis,* and* Microtus oeconomus* in Poland over a 3-year period reported significant fluctuation in *Bartonella* prevalence, attributed to changes in host and vector population densities affecting transmission efficiency [75, 76]. The minimal fluctuations observed in this study may reflect the low number of dormice sampled in certain years (2016: 16, 2021: 3) and fleas (2019: 10), and/or the absence of substantial changes in dormouse population structure. Moreover, detection in blood samples indicates ongoing bacteremia in dormice, which may not be persistent in other host species or in certain *Bartonella*–host associations.

Although overall *Bartonella* prevalence did not vary over the sampling years, the relative occurrence of the two dominant species varied significantly over time in fleas collected from dormice and nests (both *B. gliris* and *B. grahamii* subsp. *shimonis*) and dormice (only *B. gliris*). These results suggest that while species composition fluctuated over time, overall prevalence remained stable, as when one species decreased, the other increased. In fleas collected from nests, the occurrence of each species was also associated with the presence or absence of the other. In addition, the occurrence of *B. grahamii* subsp. *shimonis* in dormice was negatively correlated with host body weight, although this result is likely biased by the low number of large individuals, and further data are needed for confirmation.

### Factors influencing *Bartonella* prevalence

In Germany, edible dormice have been described as effective reservoir hosts for *Borrelia burgdorferi* s.l., largely due to their longevity (up to 9 years), high levels of ectoparasite infestation, and overall abundance [77]. These factors may at least partially explain the high prevalence of *Bartonella* spp. observed in this study.

High infestation of dormice by fleas [26] was confirmed by Bundilová (master thesis; data not published). Brettschneider et al. [78] demonstrate that only a small portion of the overall difference in *Bartonella* prevalence between *Rattus rattus* and *R. norvegicus* in South Africa can be attributed to differences in flea infestation levels. While some studies have associated higher *Bartonella* prevalence with increased ectoparasite burden [76], others have not confirmed this relationship [79, 80]. This lack of consistent relationship has been attributed to multiple transmission routes from fleas to rodents (via feces and during blood feeding), the high bacterial load required to establish infection, and differences in detection methods between rodents and fleas [80]. Therefore, part of the *Bartonella* prevalence observed in this study may be explained by high ectoparasite infestation levels. Because fleas frequently move between hosts and do not maintain long-term associations with individual dormice, we did not analyze host–vector infection pairings at the individual level. The 90% prevalence detected in fleas collected from dormice closely matched the 88% prevalence detected in dormice themselves. A similar pattern was observed in *Gerbillus andersoni* in Israel, where prevalence reached 75% and 71% in rodents and fleas, respectively [67]. In contrast, a study in Lithuania reported lower prevalence in squirrels (56%) compared to their *C. sciurorum* fleas (39%), [61].

Previous studies have reported higher *Bartonella* prevalence in juvenile rodents (*M. glareolus*, *M. oeconomus*) compared to adults. This pattern has been attributed to either infection clearance and development of immunity with age, or increase mortality of infected adults, with immunological naïveté of juveniles also considered a contributing factor [76, 79, 81]. However, the lack of a difference in *Bartonella* prevalence between age groups in this study suggests a high level of dormouse adaptation to *Bartonella* infection, with no apparent mechanism for acquired resistance or infection clearance in older animals. The similar prevalence in juveniles and adults also implies the possibility of congenital transmission, as described for *Sigmodon hispidus* and *Peromyscus leucopus* in the USA [82].

### Methods for the detection of *Bartonella* spp.

Samples were analyzed using independent nested PCR assays targeting four loci, complemented by bacteria cultivation, to ensure accurate detection of *Bartonella* infection. Among the PCR loci, *gltA* and ITS performed best overall in terms of sensitivity (Table. 2) and polymorphism (Table. S8), with ITS also offering cost-effectiveness due to shorter amplicon length differences. Nevertheless, each PCR protocol, in combination with cultivation, detected positive samples or mixed infections in cases that would otherwise have been missed by the other methods.

Previous studies have reported *Bartonella* prevalence of up to 90% in various rodent species and up to 70% in their associated fleas, depending not only on host and vector ecology but also on the detection methods employed. Screening of free-living animals commonly utilizes a range of approaches, from conventional and real-time PCR targeting multiple loci to bacterial cultivation [9, 80, 83, 84]. For example, a high prevalence (90%) in *Onychomys leucogaster* in the USA was detected using cultivation combined with single-round PCR targeting *gltA* [83]. These findings demonstrate that high prevalence can be identified even with simpler methods—or by combining approaches—and may be influenced by additional factors such as higher bacteremia levels in the studied host species or more efficient cultivation and sampling protocols.

The detection rate of mixed infections based on analysis of individual PCR loci (detected by the presence of clear double peaks) ranged from 2–32%. When results from all methods (different PCR assays and cultivation) were combined, the overall prevalence of mixed infection increased to 53%, indicating that both dormice and fleas are capable of harboring multiple *Bartonella* species simultaneously. A comparable prevalence (46%) has been reported in *Gerbillus* spp. in Israel, where PCR assays targeting *gltA, rpoB*, and ITS were applied in combination with cultivation [84]. These findings highlight the importance of using multiple loci and/or culture for accurate detection of mixed infections.

In contrast, *Bartonella* prevalence detected by cultivation was substantially lower, reaching only 60% in dormice blood samples and 22% in fleas collected from dormice and 7% in fleas collected from nests, compared with the overall PCR-based prevalence 88%, 90% and 40%, respectively. This reduced detection success was largely attributable to frequent contamination by fast-growing bacteria, which overgrew the slow-growing *Bartonella* in 27% of blood samples and 49% of flea samples. In addition, the lower prevalence observed by cultivation may partly reflect the fact that only viable bacteria are detected using this method. Nevertheless, we recommend inclusion of cultivation as a complementary approach, as it not only supports PCR-based detection but also enables the recovery of bacterial isolates for subsequent phenotypic and genotypic characterization.

### *Bartonella* infection in different flea life stages

Fleas of three life stages were analyzed to investigate potential transmission routes of *Bartonella* spp. Overall *Bartonella* prevalence was 31% in established adult fleas, 30% in larvae, and 17% in newly emerged adults. The detection of *Bartonella* in larvae suggests possible vertical transmission; however, the design of our experiment did not allow a certain conclusion, as to whether the larvae became infected transovarially/transstadially or via the feces of infected adult fleas, as described in previous studies [74]. Transovarial transmission to larvae cannot be ruled out, although this route has not been conclusively demonstrated, with only contradictory evidence available [85, 86]. Such discrepancies may reflect potential species-specific differences in transovarial transmission of *Bartonella* spp. in fleas [87].

Our results suggest the possibility of transstadial perpetuation from infected larvae to newly emerged adult fleas. However, the cultivation of *Bartonella* spp. from these samples was unsuccessful due to contamination overgrowth, and thus it remains unclear whether the bacteria detected by PCR were viable. Moreover, it is also possible that newly emerged adults retain or acquire detectable *Bartonella* DNA during pupation without being truly infected or harboring viable, culturable bacteria.

Unexpectedly, no statistically significant differences were observed in *Bartonella* prevalence among flea life stages. This result may be influenced by the limited number of dormice nests analyzed (*n* = 12) and the low number of fleas per nest (in some cases, fewer than 10), which may have led to high variability in prevalence between nests and flea stages. Additional observations are, therefore, required to confirm this finding.

Nevertheless, the substantial prevalence (17%; Table. S3) of *Bartonella*-positive newly emerged adult fleas suggests that transstadial perpetuation of *B. gliris* and *B. grahamii* subsp. *shimonis* in *C. sciurorum* fleas is possible and indicates that they could persist within flea populations for extended periods without relying on vertebrate hosts to maintain infection. Similarly, *Bartonella* DNA detected in pupae and winged (i.e., unfed, host-seeking) adults of *Lipoptena* spp. keds strongly support their suspected vector role for several *Bartonella* spp. (e.g., [88]).

### Microbiome of the flea gut and its association with *Bartonella*

This study provides the first detailed characterization of the gut microbiome of the squirrel flea, *Ceratophyllus sciurorum*, demonstrating a relatively rich microbiome composition using two independent methods: cultivation on different media and nanopore sequencing. Our results also reveal a strong and reproducible association between *Bartonella* infection and the structure of the flea gut microbiome. In naturally infected *C. sciurorum*, *Bartonella* spp. dominated the gut bacterial community, whereas uninfected fleas displayed more diverse microbiomes, typically enriched with *Staphylococcus* and other environmental or host-associated taxa. The lower number of reads and observed OTUs in the digestive tract of individual *Bartonella*-negative fleas may suggest a low bacterial load in comparison, especially considering that the two other taxa consistently dominant in flea microbiomes, *Rickettsia* and *Wolbachia*, were not present in the tested *C. sciurorum*.

While these patterns are striking, it remains unclear whether *Bartonella* actively displaces other gut bacteria, whether certain microbiome configurations facilitate *Bartonella* establishment, or whether both patterns reflect underlying differences in flea physiology, age, or blood–meal history. Nevertheless, the consistency of microbiome differences across independent experiments suggests that *Bartonella* infection is embedded within broader ecological interactions in the flea gut. Similar pathogen-associated microbiome shifts have been reported in other arthropod vectors, including ticks and mosquitoes, supporting the notion that successful vector-borne pathogens often coexist with, or contribute to, alternative microbiome states within their vectors [33–35]. From an ecological perspective, our findings emphasize that fleas should not be viewed as passive carriers of *Bartonella*, but rather as dynamic microbial ecosystems in which pathogen presence is tightly linked to community-level microbial patterns.

In contrast, *Bartonella*-negative fleas are dominated by *Staphylococcus* spp., representing a distinct alternative microbiome state that may reflect environmental acquisition, larval-stage colonization, or host-derived influences [36, 37, 89]. However, some environment-associated bacteria, such as staphylococci and enterobacteria, may also originate from the flea surface. While fleas were surface-disinfected with ethanol and dissected to lessen the impact of surface-bound bacteria, as in [35, 90, 91], some authors recommend a more aggressive washing solution containing bleach to fully eliminate surface contaminants from arthropods [92–94].

Metabarcoding detected *Bartonella* in both the gut and the body of the fleas, although the proportion was larger in the gut. While we cannot rule out cross-contamination during dissection, Bartonella DNA has previously been detected in the flea hemolymph and ovaries [85].

Technical limitations, such as uneven sequencing depth in the first experiment, may have masked more subtle effects of host sex or gut compartment on microbial diversity. Nevertheless, the consistency of major patterns across independent experiments strengthens our conclusions.

Overall, these findings underscore the importance of considering the flea microbiome when studying *Bartonella* transmission. Integrating microbiome data into epidemiological and experimental models will be crucial for a mechanistic understanding of flea-borne bartonellosis [91, 95–97].

## Conclusions

By integrating long-term ecological data with vector infection dynamics and microbiome profiling, this study provides a comprehensive view of *Bartonella* transmission in a natural rodent–flea system. The consistently high prevalence observed in dormice and their fleas, together with evidence suggestive of vertical transmission and transstadial perpetuation, indicates that flea populations may be able to maintain *Bartonella* over extended periods, even in the absence of continuous contact with vertebrate hosts.

Flea gut microbiome analyses add an important ecological dimension to this framework, revealing that *Bartonella* infection is associated with pronounced shifts in microbial community structure. Although causal relationships cannot be inferred, these patterns underscore the relevance of vector-associated microbiomes as integral components of flea-borne pathogen ecology.

Overall, the *Glis glis*–*Ceratophyllus sciurorum*–*Bartonella* system represents a robust natural model for studying flea-borne zoonoses, illustrating how long-term host–vector interactions and microbial context jointly shape pathogen transmission in the wild.

## Supplementary Information

Supplementary Material 1. Locations of the 20 sampling sites (**B**; the red circle highlights the three sites with the highest sampling intensity) within the study area (**A**; red dot) near Dlouhá Loučka, Olomouc district, Czech Republic

Supplementary Material 2. Scheme describing different steps of processing of samples taken from dormice and nest boxes, with the number of samples used for various parts of the analysis

Supplementary Material 3. Prevalence of *Bartonella grahamii* subsp. *shimonis* (shades of blue) and *B. gliris* (shades of red), determined using a combination of PCR assays and cultivation in *Bartonella*-positive samples. Samples with mixed infections were counted for all species detected, and unidentified genotypes (e.g., short sequences did not allow more detailed identification, or the *Bartonella* species was detected by *ftsZ-*PCR which did not enable genotype discrimination) are indicated as “un”

Supplementary Material 4.Representative images of the three OTUs with the highest sequence counts isolated from cultures and their occurrence on (from left) blood, chocolate, and Tryptic Soy agar. The most abundant OTU for the genera *Staphylococcus* (**A**), *Mammaliicoccus* (**B**), and *Brevibacterium* (**C**) are shown. Green arrows indicate the corresponding colonies

Supplementary Material 5.Relative frequencies of dominant taxa in pools of flea gut samples (F = female, M = male; sum = summer, win = winter; m = midgut, h = hindgut). Taxa with relative abundance of < 1% are summarized in the Other category

Supplementary Material 6.Principal coordinates analysis of the microbiome of pooled flea samples using the Weighted UniFrac Distance metric. Individual points represent pools that were *Bartonella*-positive or negative by PCR (shape) of male and female fleas (color). The analysis also incorporates the Shannon diversity index

Supplementary Material 7.Relative frequencies of dominant taxa in individual flea gastrointestinal tract (GIT) and body samples from individual male (M) and female (F) fleas. Taxa with relative abundance of < 1% are summarized in the Other category

Supplementary Material 8.Principal coordinates analysis of the flea microbiome of individual fleas using the Weighted UniFrac Distance metric. Individual points represent gastrointestinal tract (GIT) or body samples (shape) and their *Bartonella* infection status (color). The analysis also incorporates the Shannon diversity index

Supplementary Material 9.Schematic representation of sequence differences among *Bartonella grahamii* subsp. *shimonis* genotypes (1–4) obtained by PCR targeting *gltA*, using both short (nested PCR) and long (one-step PCR) amplicons; for genotype 4, only the short amplicon is available; genotypes 5 and 6 were distinguished based on *rpoB*

Supplementary Material 10.Schematic representation of sequences of (A) *B. grahamii* subsp. *shimonis* genotypes 1, 2, and 3 and B: *B. gliris* genotypes 1 and 2, obtained by PCR targeting ITS using both short (nested PCR) and long (single-step PCR) amplicons, comparing the differences between genotypes

Supplementary Material 11.

## Data Availability

Data are provided within the manuscript or supplementary information files. The data used in this study are available at 10.6084/m9.figshare.31189357. The R code used in this study is available on GitHub at https://github.com/Klimesobe/Bartonella-in-fleas-and-dormice.git.
