# Supplementary material for: Bartonella transmission and gut microbiome dynamics in Ceratophyllus sciurorum fleas and their edible dormouse hosts (Glis glis)
Source: Parasit Vectors. 2026 Jun 11;19:337. doi: 10.1186/s13071-026-07494-y (PMC13479981; doi:10.1186/s13071-026-07494-y)
Supplement: Supplementary file 1 [file 13071_2026_7494_MOESM11_ESM.docx]

**SUPPLEMENTARY TABLES:**

**Table S1.** The protocols for nested PCR of the loci *gltA*, *rpoB, ftsZ,* and ITS outline the type of PCR, the number of cycles, the temperatures for different cycles, and the specific time.

| **Loci** | ***gltA*** | | | ***rpoB*** | | | ***ftsZ*** | | | **ITS** | | |
| --- | --- | --- | --- | --- | --- | --- | --- | --- | --- | --- | --- | --- |
| **PCR type** | **No. Cycles** | **Temp.** | **Time** | **No. Cycles** | **Temp.** | **Time** | **No. Cycles** | **Temp.** | **Time** | **No. Cycles** | **Temp.** | **Time** |
| PCR 1 | 1x | 95 | 5 min | 1x | 94 | 2 min | 1x | 94 | 5 min | 1x | 95 | 5 min |
|  | 40x | 95 | 30 s | 35x | 94 | 30 s | 35x | 94 | 30 s | 35x | 94 | 45 s |
|  |  | 53 | 30 s |  | 56 | 30 s |  | 60 | 30 s |  | 56 | 45 s |
|  |  | 72 | 1 min |  | 72 | 1 min |  | 72 | 1 min |  | 72 | 45 s |
|  | 1x | 72 | 10 min | 1x | 72 | 2 min | 1x | 72 | 5 min | 1x | 72 | 10 min |
| PCR 2 | 1x | 95 | 5 min | 1x | 94 | 5 min |  |  |  | 1x | 95 | 45 s |
|  | 25x | 95 | 30 s | 35x | 94 | 30 s |  |  |  | 25x | 94 | 45 s |
|  |  | 56 | 30s |  | 55 | 30s |  |  |  |  | 58 | 45 s |
|  |  | 72 | 1 min |  | 72 | 1 min |  |  |  |  | 72 | 45 s |
|  | 1x | 72 | 10 min | 1x | 72 | 5 min |  |  |  | 1x | 72 | 10 min |

**Table S2.** Primers for the loci *gltA, rpoB, ftsZ,* and ITS were used for sample amplification and sequencing.

| **Locus** | **PCR Type** | **Primer name** | **Sequence 5’–3’** | **References** |
| --- | --- | --- | --- | --- |
| *gltA* | PCR 1 | 443f | GCTATGTCTGCATTCTATCA | Birtles & Raoult 1996 [98] |
|  |  | 1210R | GATCYTCAATCATTTCTTTCCA | Billeter et al. 2011 [99] |
|  | PCR 2 | 443f | GCTATGTCTGCATTCTATCA | Birtles & Raoult 1996 [98] |
|  |  | 781R | CCACCATGAGCTGGTCCCC | Sofer et al. 2015 [100] |
| *rpoB* | PCR 1 | 1400F | CGCATTGGCTTACTTCGTATG | Renesto et al. 2001 [101] |
|  |  | 2300R | GTAGACTGATTAGAACGCTG |  |
|  | PCR 2 | 1596F | CGCATTATGGTCGTATTTGTCC | Paziewska et al. 2011 [102] |
|  |  | 2300R | GTAGACTGATTAGAACGCTG | Renesto et al. 2001 [101] |
| *ftsZ* | PCR 1 & 2 | F | CATATGGTTTTCATTACTGCYGGTATGG | Paziewska et al. 2011 [102] |
|  |  | R | TTCTTCGCGAATACGATTAGCAGCTTC |  |
| ITS | PCR 1 | H56s | GGGGAACCTGTGGCTGGATCAC | Maggi & Breitschwerdt 2005 [103] |
|  |  | 983as | TGTTCTYACAACAATGATGATG |  |
| ITS | PCR 2 | H493as | TGAACCTCCGACCTCACGCTTATC | Maggi & Breitschwerdt 2005 [103] |
|  |  | 321s | AGATGATGATCCCAAGCCTTCTGG |  |

**Table S3.** Numbers of tested and ITS-PCR *Bartonella*-positive primary adult fleas, larvae, and newly emerged adult fleas from dormice nests.

| **Nest** | **Primary adult fleas** | | **Larvae** | | **Newly emerged adult fleas** | |
| --- | --- | --- | --- | --- | --- | --- |
|  | **Pos./Overall** | **Positive [%]** | **Pos./Overall** | **Positive [%]** | **Pos./Overall** | **Positive [%]** |
| H3 | 1/10 | 10 | 0/3 | 0 | 4/25 | 16 |
| H4 | 2/10 | 20 | 2/7 | 29 | 3/4 | 75 |
| H6 | 5/10 | 50 | 6/10 | 60 | 2/11 | 18 |
| H8 | 1/10 | 10 | 0/8 | 0 | 0/10 | 0 |
| H9 | 8/11 | 73 | – | – | 0/4 | 0 |
| H9b | 0/3 | 0 | 0/4 | 0 | – | – |
| H10 | 2/10 | 20 | 9/10 | 90 | 4/11 | 36 |
| H11 | 0/10 | 0 | 3/4 | 75 | 5/10 | 50 |
| H12 D | 5/6 | 83 | 0/7 | 0 | 1/10 | 10 |
| H12 H | – | – | – | – | 0/10 | 0 |
| H13 | 1/4 | 25 | 1/10 | 10 | 3/10 | 30 |
| H15 | 8/22 | 36 | 1/10 | 10 | 0/21 | 0 |
| Overall | 33/106 | 31 | 22/73 | 30 | 22/126 | 17 |

**Table S4.** Generalized linear mixed-effect models assessing the effect of multiple variables on the detected bartonellae prevalence in individual dormice (n = 201), fleas sampled from dormice (n = 410 with sex, n = 510 without sex), or fleas sampled from nests (n = 187) tested by type II Wald Chi-square test. Values highlighted by * indicate P values lower than 0.05.

| **Samples** | **Random effect** | **Fixed effects** | **Levels** | **χ2** | **D.F.** | **P** |
| --- | --- | --- | --- | --- | --- | --- |
| Dormice | - | Weight | 23-190 | 0.08 | 1 | 0.78 |
|  |  | Age | Juvenile/Adult | 1.6 | 1 | 0.2 |
|  |  | Sex | F/M | 0.006 | 1 | 0.94 |
|  |  | Year | 2016-2021 | 3.6 | 5 | 0.61 |
| Fleas from dormice | Dormice ID | Sex | F/M | 2.12 | 1 | 0.15 |
|  |  | Year | 2017-2021 | 6.46 | 2 | 0.04* |
|  | Dormice ID | Year | 2017-2021 | 8.21 | 4 | 0.08 |
| Fleas from nests | Nests ID | Year | 2019-2021 | 1.92 | 2 | 0.38 |

**Table S5.** Generalized linear mixed-effect models showing the effect of multiple variables on the detected prevalence of *B. gliris* and *B. grahamii* subs. *shimoni* in individual dormice (n = 201), fleas sampled from dormice (n = 510), or fleas sampled from nests (n = 187) tested by type II Wald Chi-square test. Variables were tested in various order. Values highlighted by * indicate P values lower than 0.05.

| **Samples** | **Variable** | **Random effect** | **Fixed effects** | **Levels** | **χ2** | **D. F.** | **P** |
| --- | --- | --- | --- | --- | --- | --- | --- |
| Dormice | *B. g.* subs. *shimonis* | - | *B. gliris* | 0/1 | 3.85 | 1 | 0.05 |
|  |  |  | Weight | 23-190 | 8.33 | 1 | 0.004* |
|  |  |  | Age | Juvenile/Adult | 0.69 | 1 | 0.41 |
|  |  |  | Sex | F/M | 0.02 | 1 | 0.89 |
|  |  |  | Year | 2016-2021 | 4.55 | 5 | 0.47 |
|  | *B. gliris* | - | *B. g.* subs. *shimonis* | 0/1 | 3.73 | 1 | 0.05 |
|  |  |  | Weight | 23-190 | 0.82 | 1 | 0.37 |
|  |  |  | Age | Juvenile/Adult | 1.29 | 1 | 0.25 |
|  |  |  | Sex | F/M | 0.21 | 1 | 0.65 |
|  |  |  | Year | 2016-2021 | 14.3 | 5 | 0.01* |
| Fleas from dormice | *B. g.* subs. *shimonis* | Dormice ID | *B. gliris* | 0/1 | 2.88 | 1 | 0.09 |
|  |  |  | Year | 2017-2021 | 26.28 | 4 | <0.01* |
|  | *B. gliris* | Dormice ID | *B. g.* subs*. shimonis* | 0/1 | 3.2 | 1 | 0.07 |
|  |  |  | Year | 2017-2021 | 22.19 | 4 | <0.01* |
| Fleas from nests | *B. g.* subs. *shimonis* | Nests ID | *B. gliris* | 0/1 | 16.5 | 1 | <0.01* |
|  |  |  | Year | 2019-2021 | 6.8 | 2 | <0.03* |
|  | *B. gliris* | Nests ID | *B. g.* subs*. shimonis* | 0/1 | 16.5 | 1 | <0.01* |
|  |  |  | Year | 2019-2021 | 14.42 | 2 | <0.01* |

**Table S6.** Prevalence of *Bartonella grahamii* subsp. *shimonis*, *B. gliris*, and mixed infections among Bartonella-positive samples as identified by five individual detection methods (*gltA*, *rpoB*, *ftsZ*, ITS, and cultivation) and by an overall assessment combining results from all approaches. Cultivation was performed on a subset of samples.

| **Prevalence**  **[%]** | ***B. grahamii subsp. shimonis*** | ***B. gliris*** | **Mixed infections** |
| --- | --- | --- | --- |
| *gltA* | 50 | 24 | 24 |
| *rpoB* | 65 | 15 | 2 |
| *ftsZ* | 28 | 38 | 32 |
| ITS | 30 | 37 | 32 |
| Cultivation | 35 | 39 | 26 |
| Overall | 25 | 20 | 53 |

**Table S7.** Bacterial genera identified in flea microbiome cultures, with the corresponding number of OTUs and tested colonies (i.e., sequence counts).

| **Genus** | **OTUs** | **Colonies tested** |
| --- | --- | --- |
| *Staphylococcus* | 11 | 134 |
| *Arthrobacter* | 7 | 18 |
| *Brevibacterium* | 5 | 50 |
| *Microbacterium* | 5 | 16 |
| *Brachybacterium* | 4 | 29 |
| *Streptomyces* | 4 | 6 |
| *Mamaliicoccus* | 3 | 42 |
| *Sphingobacterium* | 2 | 6 |
| *Dietzia* | 2 | 4 |
| *Micrococcus* | 2 | 4 |
| *Bacillus* | 2 | 3 |
| *Sporosarcina* | 2 | 3 |
| *Rahnella* | 1 | 26 |
| *Citrobacter* | 1 | 8 |
| *Nocardiopsis* | 1 | 7 |
| *Carnobacterium* | 1 | 6 |
| *Advenella* | 1 | 5 |
| *Kocuria* | 1 | 5 |
| *Frondihabitans* | 1 | 4 |
| *Pollutimonas* | 1 | 3 |
| *Moraxella* | 1 | 2 |
| *Psychrobacter* | 1 | 2 |
| *Rothia* | 1 | 2 |
| Uncultured bacterial clone^[1]^ | 1 | 2 |
| Uncultured beta proteobacterium^[2]^ | 1 | 2 |
| *Cedecea* | 1 | 1 |
| Gamma proteobacterium^[3]^ | 1 | 1 |
| *Chryseobacterium* | 1 | 1 |
| *Lysobacter* | 1 | 1 |
| *Neobacillus* | 1 | 1 |
| *Novilysobacter* | 1 | 1 |
| *Ochrobactrum* | 1 | 1 |
| *Paenochrobactrum* | 1 | 1 |
| *Rhodanobacter* | 1 | 1 |
| *Stenotrophomonas* | 1 | 1 |
| Uncultured bacterial clone^[4]^ | 1 | 1 |
| Uncultured bacterial clone^[5]^ | 1 | 1 |
| *Myroides* | 1 | 1 |
| *Pusillimonas* | 1 | 1 |
| *Virgibacillus* | 1 | 1 |

**Table S8.** Genotypic resolution of *Bartonella gliris* and *Bartonella grahamii* subsp. *shimonis* by (nested) PCR assays targeting four loci. The table summarizes the number of genotypes of *Bartonella gliris* and *Bartonella grahamii* subsp. *shimonis* distinguished by individual (nested) PCR protocols targeting *gltA*, *rpoB*, *ftsZ*, and ITS.

|  | *B. grahamii* subsp. *shimonis* | *B. gliris* |
| --- | --- | --- |
| *gltA* | 4 | 4 |
| *rpoB* | 3 | 3 |
| *ftsZ* | 1 | 1 |
| ITS | 3 | 2 |

**Table S9:** Number of reads, OTUs and taxa for nanopore metabarcoding analyses.

|  |  | Pooled samples | Individual fleas |
| --- | --- | --- | --- |
|  | Reads | 443 654 | 129 469 |
|  | High quality reads | 83 858 | 84 411 |
|  | OTUs | 1 429 | 1 250 |
|  | OTUs after decontamination | 1 419 | 1 232 |
|  | Rarefaction treshold | 255 | 715 |
|  | OTUs after rarefaction | 453 | 959 |
| Identified taxa | Phylum | 9 | 12 |
|  | Class | 15 | 15 |
|  | Order | 39 | 37 |
|  | Family | 67 | 49 |
|  | Genus | 85 | 57 |

**Table S10:** Alpha diversity analyses of the bacterial composition of flea samples. F = female, M = male; sum = summer, win = winter; m = midgut, h = hindgut. GIT = gastrointestinal tract. Numbers represent the ID of each pool/flea.

|  | Observed richness | Shannon index | Faith's phylogenetic diversity | Simpson's dominance |
| --- | --- | --- | --- | --- |
| **Pooled samples** | | | | |
| M4-sum-h | 54 | 4.738 | 3.253 | 0.933 |
| F5-sum-h | 52 | 4.707 | 3.387 | 0.933 |
| F6-sum-m | 65 | 5.403 | 3.711 | 0.967 |
| F6-sum-h | 39 | 4.163 | 1.690 | 0.888 |
| M6-sum-m | 37 | 3.276 | 1.578 | 0.768 |
| F3-sum-m | 31 | 3.436 | 1.605 | 0.841 |
| F3-sum-h | 43 | 3.169 | 3.787 | 0.694 |
| M3-sum-h | 24 | 2.834 | 2.132 | 0.719 |
| F2-win-h | 94 | 5.998 | 4.510 | 0.978 |
| M2-win-m | 74 | 5.346 | 5.097 | 0.954 |
| M2-win-h | 43 | 4.119 | 5.716 | 0.897 |
| **Individual fleas** | | | | |
| M02-GIT | 75 | 4.692 | 1.758 | 0.937 |
| F05-GIT | 47 | 3.410 | 1.416 | 0.772 |
| F12-GIT | 51 | 3.393 | 1.879 | 0.826 |
| M13-GIT | 84 | 4.797 | 3.419 | 0.937 |
| M14-GIT | 49 | 2.157 | 1.968 | 0.529 |
| F15-GIT | 63 | 3.527 | 2.241 | 0.829 |
| F20-GIT | 55 | 3.298 | 1.343 | 0.807 |
| F21-GIT | 60 | 3.066 | 2.383 | 0.719 |
| M02-body | 78 | 4.615 | 2.512 | 0.933 |
| F03-body | 112 | 5.165 | 7.365 | 0.927 |
| M04-body | 75 | 4.154 | 2.333 | 0.864 |
| M08-body | 68 | 3.953 | 2.901 | 0.857 |
| M11-body | 120 | 5.876 | 5.118 | 0.966 |
| F12-body | 9 | 0.245 | 1.107 | 0.052 |
| F15-body | 112 | 4.689 | 5.355 | 0.903 |
| M18-body | 139 | 5.940 | 5.261 | 0.968 |
| F20-body | 67 | 3.574 | 2.624 | 0.824 |
| F21-body | 85 | 4.200 | 3.241 | 0.875 |
| F24-body | 115 | 5.434 | 3.488 | 0.951 |

**Table S11:** Differentiation of individual genotypes based on the target loci used. Light grey – genotypes successfully cultivated; * found in a single dormouse sample only; # detected in a single flea sample only.

| **Genotype designation** | **Sequences obtained by different PCR targeted loci** | | | | | | |
| --- | --- | --- | --- | --- | --- | --- | --- |
|  | ***gltA* short sequence** | ***gltA* long sequence** | ***rpoB* short sequence** | ***rpoB* long sequence** | **ITS short sequence** | **ITS long sequence** |  |
| *B. g. shimonis* 1 | *B. g. s. 1/2* | *B. g. s. 1* | *B. g. s. 1/3* | *B. g. s. 1* | *B. g. s. 1* | *B. g. s. 1* |  |
| *B. g. shimonis* 2 | *B. g. s. 1/2* | *B. g. s. 2* | *B. g. s. 2* | *B. g. s. 2* | *B. g. s. 2/3* | *B. g. s. 2* |  |
| *B. g. shimonis* 3 | *B. g. s. 3* | *B. g. s. 3* | *B. g. s. 1/3* | *B. g. s. 3* | *B. g. s. 2/3* | *B. g. s. 3* |  |
| *B. g. shimonis* 4* | *B. g.* s*.* 4 | - | - | - | - | - |  |
| *B. gliris* 1 | *B. gliris* 1 | *B. gliris* 1 | *B. gliris* 1*/2* | *B. gliris* 1*/2* | *B. gliris* 1*/2* | *B. gliris* 1 |  |
| *B. gliris* 2 | *B. gliris* 2 | *B. gliris* 2 | *B. gliris* 1*/2* | *B. gliris* 1*/2* | *B. gliris* 1*/2* | *B. gliris* 2 |  |
| *B. gliris* 3 | *B. gliris* 3 | - | - | - | - | - |  |
| *B. gliris* 4 *#* | *B. gliris* 4 | - | - | - | - | - |  |
| *B. gliris* 5 *#* | - | - | *B. gliris* 5 | - | - | - |  |
| *B. gliris* 6 *#* | - | - | *B. gliris* 6 | - | - | - |  |

1. KM870352
2. AJ318192
3. FJ164057.1
4. MF583881.1 nebo MF047082.1
5. AY268339.1
